# Fictions and frictions: Promises, transaction costs and the innovation of network technologies

**DOI:** 10.1177/0306312719838339

**Published:** 2019-03-18

**Authors:** Udo Pesch, Georgy Ishmaev

**Affiliations:** Faculty of Technology, Policy & Management, Delft University of Technology, Delft, The Netherlands

**Keywords:** blockchain, decentralized market places, fintech, network technologies, technical promises, transaction costs

## Abstract

New network technologies are framed as eliminating ‘transaction costs’, a notion first developed in economic theory that now drives the design of market systems. However, the actual promise of the elimination of transaction costs seems unfeasible, because of a cyclical pattern in which network technologies that make that promise create processes of institutionalization that create new forms transaction costs. Nonetheless, the promises legitimize the exemption of innovations of network technologies from critical scrutiny.

## Introduction

Promises about the capacities of new technologies are an important driver for technological development, helping to mobilize resource bases (Van Lente and Rip, 1998). In the realm of network technologies – which we understand broadly to mean technical infrastructures that allow the transport of elements such as electricity, information or waste between different physical locations – these promises often revolve around the ambition to decrease or even cancel out transaction costs. This ambition finds its root in economic theory, which presents transactions costs as frictions that hamper the economy. A particularly strong example of claims pertaining to the emergence of ‘frictionless’ markets can be found in the field of financial technology (‘fintech’), especially with the recent development of distributed ledger (DL) or blockchain technologies (Davidson et al., 2016a; Hazard et al., 2016).^1^ Though these technologies are still very much in development, we focus on them to reflect on the dynamic interplay between promises, innovations and the development of new and unforeseen transaction costs. We aim to show that the focus on the eradication of transaction costs, first, allows technological development to be exempted from public scrutiny, and, second, narrows the scope of societal and economic concerns to an undesirable extent as new societal and economic asymmetries emerge. Its normative claim is that promises about the reduction of transaction costs should be not be taken at face value, but they should be made into subject of public reflection and deliberation.

The case of network technologies can be seen as a strong example of how economic theory is *performative* for economic markets (Birch, 2017; Callon, 2010; MacKenzie, 2003), in the sense that correspondences ‘between economic theory and economic reality are not discovered but built’ (Breslau, 2013). This paper will examine how this performativity of economics plays out with regards to the notion of ‘transaction costs’, a concept originally introduced to explain why markets include institutional structures such as bureaucratic organizations, but since then taken as a real obstacle for further economic progress. This shift from theory to practice has inspired the development of new technologies that could conquer these costs.

‘Decentralized markets’ that have followed the development of blockchain technologies are surrounded by the most ambitious promises to date.^2^ Not only do such blockchain-based solutions explicitly target transaction costs, they often define various societal and economic problems as articulations or results of transaction costs, bypassing the possibility of other definitions, while paradoxically creating new and new forms of transaction costs. We identify some new transactions costs and conclude that the future social costs of ‘fintech’ developments are still largely unarticulated, arguably precisely because it is assumed that the frictionless market will mitigate these costs.

We emphasize a need for broader discussions regarding underlying normative-theoretical assumptions, if we want to have new financial technology aligned with societal demands and restrictions. The article aspires to provide starting points for a broader discussion that should pertain to the following issues: the new responsibilities for technology developers, regulators and researchers, the widening of the scope of societal concerns relevant to the technology, and the repercussions of new asymmetries of information and power.

## Institutions, transaction costs and technical promises

In the field of institutional economics, transaction costs are connected to institutions, the latter of which Nobel Prize-winner Douglas North (1989) defines as ‘rules, enforcement characteristics of rules, and norms of behavior that structure repeated human interaction’. Another Nobel laureate in economics, Ronald Coase (1937, 1960), argues that the function of institutions is to reduce transaction costs, those costs of engaging in economic transactions. If two agents have the same repertoire of available courses of action, they do not have to make calculations about *all* possible courses of action, thus making it possible to economize on negotiation costs.

The emergence of the idea of transaction costs can be connected to the institutional innovation of modern hierarchical organizations, which, according to Chandler (1977), finds it root in the construction of the railroad system in the United States. Before the introduction of the railroad companies in the 1840s, the localized nature of production allowed for relatively traditional forms of organization. The control over this extensive infrastructure necessitated a new type of business enterprise, which was based on the division into multiple organizational units that had to be managed separately.

The railroad is an exemplary case of a technical system. Such a system involves the connections of different parts, and as it evolves it increases in strength; the connections between the parts become tighter, making it difficult to bypass the system as a whole (Hughes, 1983). The fact that the concept of transaction costs emerged in the context of network technologies has major ramifications for later understandings of this concept. Transaction costs came to be understood as most often or centrally concerning *information flows*. As management spread over time and space, information transfer became indispensable. Reduction of the ‘price’ of information flows came to be seen as the main route forwards in conquering transaction costs. The promise of the capacities of new technology had to be directed towards their ability to reduce the ‘price’ of information flows.

This brings us to a second set of questions about theory, relating to the drivers of institutional and technical change. North (1996) makes the suggestion that such change follows from the *beliefs* of entrepreneurs introducing institutional or technological innovation in order to improve their competitive positions. This starting point is also recognizable in the work of evolutionary economists and innovation scholars, who explain how technology producers are motivated by beliefs about the qualities of technologies-to-be (Garud and Rappa, 1994; Nelson and Winter, 1977).

In this, two future-oriented mechanisms stand out in their constitutive role for innovation processes. The first of these relates to the making of promises, necessary to obtain investments. Technology developers have to apply for discursive, material and institutional resources (Pesch et al., 2017; Raven et al., 2015). This means that technology developers have to convince actors controlling resources of the benefits of investments in the new technology (Dignum, 2013; Van Lente, 1993; Van Lente and Rip, 1998). The second prerequisite relates to the coordination of activities that is facilitated by shared expectations about the capacities of a future technology. In general, the development of contemporary technologies relies on the efforts of distributed networks (Elzen et al., 1996; Pesch, 2015). Shared expectations and visions about the functioning of the technology allow these social networks to have a concerted approach to the creation of new technologies. Also here, performativity plays a crucial role, as promises and expectations can be said to contribute to their own enactment (Borup et al., 2006; Brown and Michael, 2003; Selin, 2008). In the case of conquering transaction costs through network technology development, this performativity of promises and beliefs appears to dovetail with the performativity of economic theory. It needs to be emphasized that beliefs about technical futures are not value-free; quite the opposite, they pertain to ‘sociotechnical imaginaries’ containing explicit and implicit accounts of what society is and what it should be (cf. Jasanoff and Kim, 2009).

Articulating promises explicitly in terms of the eradication of transaction costs appears to have emerged in the context of the internet. For instance, in 1995 Microsoft CEO Bill Gates wrote that the internet would allow a ‘friction-free capitalism’:

[I]f every buyer knew every seller’s price and every seller knew what every buyer was willing to pay, then everyone in the ‘market’ would be able to make fully informed decisions and society’s resources would be distributed evenly. To date we haven’t achieved Smith’s ideal because would-be buyers and would-be sellers hardly ever have complete information … The Internet will extend the electronic marketplace and become the ultimate go-between, the universal middleman … It will be a shopper’s heaven. (Gates et al., 1995)

The promise of an economy without transaction costs, which is imagined in Gates’s frictionless capitalism, can be seen as a guiding vision that motivates resources to be directed towards technology development. In the 1990s, this guiding vision might have led to the internet bubble of 2000 (Goodnight and Green, 2010), but this proved to be a temporary hiccup, followed by the creation of wireless networks, smartphones, online shops and the internet-of-things, and now DL-based ‘fintech’ applications.

As technologies are developed and implemented, they produce new problems, challenges and opportunities that may give rise to a new round of promises, motivating quests for new technological solutions (cf. Bijker, 1997), as well as giving rise to new sociotechnical practices and arrangements. In other words, a new technology elicits different forms of specialization, creating unforeseen rules and patterns of interaction.

In the context of network technologies, it is important to introduce ‘positive feedback loops’ as a special type of path-dependency (Arthur, 1989). Positive feedback loops emerge in situations where a relatively small advantage, for instance a slightly higher market share, creates a virtuous cycle that leads to the dominance of that technology. This means that instead of having negative market returns, as is conveyed in conventional economic theory, there are ‘positive market returns’.

Network technologies are especially prone to such positive returns, gaining higher market shares by being dominant. The gain may not be only an economic mechanism, as it is also taken up as a business strategy by monopolists to acquire or defend their position (Mosco, 1999). The outcome of such monopolistic tendencies is that these technologies create asymmetries: They not only tend to exclude certain issues and concerns from consideration, but also sustain inequalities with respect to who controls the technology, who has access, and which cognitive and technical skills are needed to use it.

Going back to the case of the internet, it is apparent that we have not yet witnessed the exclusion of middlemen announced by Gates. Instead, online shops and social media platforms benefit from their dominant positions in the network. The positive feedbacks that some companies enjoy give them a tremendous advantage, not just in terms of customers and visitors but above all in terms of their capacity to gather and market the information of these customers and visitors. In this, new asymmetries have been created that oppose the starting premises of an ideal-typical capitalist market instead of furthering them.

A first asymmetry pertains to the playing field that is made up of business competitors, in which a limited number of successful companies overshadow a gigantic population of small players. A second asymmetry pertains to the enormous information advantage that companies have over customers: Collecting personal data, companies acquire significant bargaining leverage, especially if the companies obscure practices of data collection and use (Acquisti et al., 2016). Another development is that the security and reliability of information often becomes compromised. Automated profiles and messages, and different types of malware create additional costs that oppose the outlines of a frictionless capitalism (Van Eeten et al., 2007). In that respect, the resolution of Coase’s problem of externalities through the reduction of transaction costs has not materialized either. On the contrary, the social costs of cybersecurity and privacy infringements appear to increase dramatically and often these are carried by those who do not benefit from technology at all. Companies profiting from the brokerages of private data essentially offload costs of data breaches onto the profiled individuals, as illustrated by the hack of ‘Equifax’ credit reporting agency (Mathews, 2017).

Another salient feature is that customers and users often lack trust in the technical interfaces they use. These users are facing a new form of transaction cost, which can, for the time being, only be paid by defecting to psychological strategies that only allow them to believe that uncertainties are reduced (Wu et al., 2014). The irony is that digital consumer behavior does not converge to more rationality. In the context of private data collection, this situation is illustrated by an observed ‘privacy paradox’ in the gap between the rational privacy preferences and actual user behavior. This gap is explained by the malleability of users’ choices regarding private data disclosures, facilitated by the intentional design of technology often aimed to invoke attitudes of (misplaced) trust (Brandimarte et al., 2013).

The theoretical notion of transactions costs has been developed in response of historical developments, and the costs have become seen as the key factor that separates the incomplete economic system from becoming a perfect market. With that, their scope has extended tremendously, spilling over into our non-economic daily activities. As internet users, individuals involuntarily get involved in personal data markets, becoming data subjects and having to bear data protection costs. At the same time, further technological progress has invoked new commercial practices and new economic routines. Processes of specialization in terms of data management and online economic entrepreneurship have induced new costs. One may say that as data streams become more efficient, controlling the societal implications of streaming data becomes increasingly demanding. We may think here of policies about privacy, cartel flows and digital crimes, but also of the digital routines that internet users deploy in order to deal with the huge information asymmetries that they are subjected to. As we show in the next section, these new social costs have not suspended further progress. On the contrary, fighting transaction costs becomes a stronger and stronger motivation for technology development, as economic aspirations become intertwined with ideological ones.

## The temptations of fintech

### Towards a Coasean utopia

The themes introduced above are important in the case of new financial technologies. Many blockchain-based fintech projects can be seen as embodiments of distributed R&D-networks *par excellence*, often being developed on open-source principles, implying a decentralized cooperation of developers. Also, their further development recruits highly ambitious promises, both of an economic and of an ideological nature (Karlstrøm, 2014). Furthermore, it can be speculated that some of the fintech-developments implicitly embrace a strong interpretation of Coase’s approach (not embraced by Coase himself) that expects social costs to be resolved by negotiation of market actors only, without any regulatory interventions (Hanly, 1992).

Whereas Coase’s idea of a world without transaction costs figured as a thought experiment aimed to highlight the importance of transaction costs for institutional design, this imaginary world has become a blueprint, a technological utopia that embraced the reduction of transaction costs as a goal in itself. While not all fintech applications assume this promise (Winkler and Matthies, 2018), it is certainly true for many blockchain-based projects (Davidson et al., 2016b). Of particular interest are ‘smart contracts’ – technologies that almost exclusively recruit the promise of the frictionless economy (Hazard et al., 2016) and decentralized marketplaces enabled by cryptocurrencies and smart contract applications. Such decentralized marketplaces may not yet have been widely adopted, but there is a growing number of projects, some of which go beyond mere proof-of-concept implementations (Klems et al., 2017; Sidhu, 2017).^3^

In the context of smart contracts and cryptocurrencies, conventional ideas about transaction costs are seen as obsolete (Buterin, 2014; Hazard et al., 2016; Szabo, 1997). First, cryptocurrencies essentially eliminate the need for a third-party authority to delineate and enforce of property rights, as these are replaced by network consensus rules that prescribe rights and obligations of network participants, and guarantee their implementations (Davidson et al., 2016a). Second, blockchain protocols, capable of encoding complex sets of rules (sometimes labeled ‘Turing complete’), allow not only ledger-keeping, but also distributed computation, prototypically implemented as smart contracts. Smart contracts, seen as a general purpose, distributed applications that can be executed on the blockchain with code and states stored in the ledger, are also capable of emulating some functional properties of legal contracts. Again, the enforcement of these contracts does not require any third party, since implementations of contractual obligations are performed on the basis of the consensus between nodes of distributed network (Buterin, 2014).

### The workings of OpenBazaar

Probably the so-far most well-documented project that involves distributed ledger technology is OpenBazaar (see https://openbazaar.org/), an ideologically driven, open-source project – though it should be noted that OpenBazaar is being developed by the for profit company OB1 (Burnham, 2015), warranting further scrutiny for the ideological component. The platform does not aim to achieve a rent business model on the basis of a technologically enabled frictionless market, but instead has the primary goal of eliminating all hidden and implicit costs, such as privacy and cybersecurity risks, associated with existing e-commerce platforms (OpenBazaar.org, 2017b). To deliver on these promises, OpenBazaar employs a number of ingenious technological solutions unified by the concept of decentralization – relying on the key enabling component of blockchain-based cryptocurrencies for payments.^4^ The marketplace itself has a database listing of goods and services is implemented on the basis of IPFS, a hypermedia peer-to-peer protocol. Unlike the standard Internet HTTP protocol that provides delivery of the content on the basis of location (specific servers), IPFS is a content-centric approach similar to BitTorrent, where content can be fractured and distributed in the peer network while remaining searchable through the hash pointers. Accordingly, all the listings on the OpenBazaar are stored in the distributed database without central servers. This brings about two critical consequences: First, there is no central database provider that can claim intermediary fees, and, second, the marketplace is potentially censor-resistant. Furthermore, there is no single centralized search engine (as of this writing there are several search engines), and any provider can freely index the marketplace data base. This means that fees associated with the information asymmetry of market participants can also be lower than in in the case of competitors like Amazon.

Another important feature of the OpenBazaar platform concerns the resolution of contracts and litigations by a number of mechanisms. At this moment, this is arguably the least novel aspect, since, as with many other existing platforms, market participants are encouraged to rely on the reputation of their counteragents, and use ‘Ricardian contracts’ rather than blockchain based smart contracts, for arbitrage.^5^ Still, some particular solutions are worthy of attention here. For instance, any participant can potentially perform the function of an arbiter in the ‘escrow’ for a small fee. An escrow (that can be implemented as a multi-signature cryptocurrency wallet) is basically a system that allows a contractual payment to be frozen until the agreement is achieved between majority of contracting parties (seller and buyer, or seller and notary about the contractual obligations). In order to avoid the collusion of interests, contracting parties can employ an extended number of independent notaries. Other potential options include double (mutual) escrows, where both parties freeze funds so that a defaulting party inevitably loses, or even smart contracts that ensure automatic implementation of contractual obligations. Still, the crucial point is that no single authority that could charge rent for the mitigation of contractual issues is present here, thus creating potential for lowered transaction costs, on litigations and contract enforcements. Currently in development is an additional layer built on Ethereum blockchain that can provide decentralized advertising on the platform, using smart contracts for the implementation of auctions for ad content spaces.^6^ Finally, the implementation of cryptocurrency payments and anonymity (TOR) network aims to eliminate transaction costs associated with privacy and cybersecurity of market participants. Using cryptocurrencies for payment, market participants can enjoy lower intermediary fees for the transfer of value operations in the absence of centralized financial intermediaries such as banks and payment systems like PayPal.

### The challenges of decentralized market places

The promises made about decentralized marketplaces such as OpenBazaar need to be qualified. A starting observation here is that, following Kaivanto and Prince (2017), promises of lower transaction costs brought by the use of cryptocurrencies – one component of future decentralized marketplaces – are more nuanced and multifaceted than is sometimes presented. New transaction costs may arise from information asymmetries between developers and users, high volatility, currencies exchange rate manipulations and serious cybersecurity risks.^7^ Moreover, these tools and instruments can bring about new forms of negative market externalities, such as the propagation of markets for illegal goods and services following the so-called darknet markets’ paradigm (hosted on the TOR network). The social impact of such markets is too broad an issue for univocal assessment, but there is little doubt that they can carry new and unexpected social costs for the non-participants of such markets.

Thus it can be argued that while these technologies may enhance the privacy of market participants (in terms of legal goods and services), they can also impose new cybersecurity and privacy risks when such platforms contribute to the propagation of trade in identity data and malware, affecting an even wider number of stakeholders. OpenBazaar developers appear to acknowledge these issues, but address them in an idealistic interpretation of the Coasean framework by expecting that various third-party providers of search engines for the marketplace will implement the appropriate policing practices (OpenBazaar.org, 2017a). Whether these solutions can be implemented is an issue for future empirical research, but it is clear that not only new transaction costs, but also market externalities are issues deserving thorough research, since mere reliance on market mechanisms and the ‘wisdom of the crowd’ may repeat the same pattern of unfulfilled promises we have seen in case of the frictionless economy.

It is also doubtful whether truly decentralized community projects that are driven by the ambition to eliminate privacy costs for users will be able to claim sufficient market share. Early studies of Initial Coin Offerings (ICOs)^8^ already suggest that established market participants, rather than small startups aiming to disrupt existing markets, may be better positioned to benefit from blockchain-based crowdfunding to support their business models (Sehra et al., 2017). Information asymmetries created by the increased complexity of blockchain technology may encourage market participants to maintain these asymmetries. For instance, there is a possibility that decentralized data marketplaces, which propagate monetization of private data via new delivery channels, could reinforce market positions of existing data brokers, while at the same time they will impose new moral costs associated with the commodification of privacy rights on society. Having said that, it is also important not to underestimate the disruptive potential of these technological systems, which can challenge existing e-commerce giants.

Transaction costs do not evaporate because of the rise of decentralized markets or other forms of fintech. Instead, these technologies create new rules and practices that induce new costs. Though it might be the case that, on the whole, the new transaction costs are lower, they also contain some rather worrying aspects. Consumers have not become more ‘rational’ in cyberspace, but increasingly appear to assume heuristics for trust, as seen in many examples of ICO funding schemes prompting crackdowns from government regulators (e.g. Choudhury, 2017). One of the threats of such unfounded forms of trust is the development of decisive power and consequent information asymmetries, effectively negating the disrupting potential of new technology and shifting promises of zero transaction costs into future timelines.

The expanding scope of motivations to tackle innovation costs also strongly influences the conceptual framework of innovation. By promoting technological development in terms of promises of ‘reducing transaction costs’ or ‘creating more trust’, the capacity to express other considerations needing to be taken into account is drastically reduced. These promises come to define the future technology, but they also come to define those who will (have to) use this technology. As this technology-imposed definition of affected actors becomes mixed with the technical complexity of new forms of information and communication technologies, a skewed distribution of knowledge and resources may further undermine the overall ethical and societal acceptability of these technologies.

## Questioning the perpetuum mobile of innovation

In this article, we have explored an intertwining of promises and technological development in the development of network technologies. This development can be sketched out as the following circular pattern:

1. Innovators promise that their new technology will lead to the reduction (or even eradication) of transaction costs.
2. A resource base, consisting of investments, institutional support and legitimacy, is created so the new technology can be further developed.
3. The societal uptake of the new technology creates new processes of institutionalization, and new forms of professional, cognitive and technical specialization emerge as responses to the development of new sociotechnical systems.
4. New forms of specialization rules set off new transaction costs and the loop will start of again.

This cyclical patterns seemingly gives rise to a ‘perpetuum mobile’ of conquering and creating transactions costs. Indeed, increases in calculative powers and interconnectedness give rise to new promises that are more far-reaching than ever before. The development of blockchain-based solutions is based on promises to eliminate transaction costs by eliminating market fees of intermediation carried out by centralized authorities. Such tempting visions of markets with zero transaction costs obscure questions about the overall desirability of the technologies and institutions involved. Added to that is the observation that, in the absence of appropriate regulations, existing market monopolies should be able to successfully colonize new distributed ledger-enabled ecosystems and institutions, so that market asymmetries are further reinforced. In those cases, the promises of decentralization and consequent social benefits may fail to materialize.

The pull of reducing transaction costs is part of a sociotechnical imaginary that is not reflected upon and that allows technology developers to evade responsibility for the moral implications of their work. As we have shown, new information technologies may come with new asymmetries of power, which necessitate not only new behavioral rules, but also new moral codes that have to address these asymmetries. Moreover, the exclusive focus on information and accompanying transaction costs might hinder a full grasp of associated societal and political problems. Indeed, digital networks are effectuations of specific social theories that in many cases have strong ideological connotations (cf. Marres, 2017). At times, actors may pose questions about the validity of these theories and ideologies, but the ways in which they shape real social interactions are not subjected to debate and scrutiny – perhaps because of an assumed separation of technology and society, and perhaps because of a fear of missing out on the opportunities offered by the new technologies.

The reluctance to intervene and scrutinize also has to do with a reliance on policies that only target negative side-effects, such as cybercrime and privacy protection, of these technologies. Policies, however, can go much further than just remedying side-effects; they can actively guide the innovation process so as to develop new technologies that are broadly desirable – especially if they are as radical as fintech and have potential to affect fundamental human rights.

Given the constitutive role of promises, it seems constructive from a democratic and ethical point of view to think about the possibilities of turning the articulation of promises and expectations in to an inclusive and deliberative process. Promises about the reduction of transaction costs should be not be taken at face value, but they should be made into subject of reflection and deliberation. In this, it is of the utmost importance not to see promises as unavoidable futures, but as strategic activities to help innovators and other stakeholders build the resource base they require to further their goals and interests. This take on the role of promises in technology development implies that new responsibilities have to be taken up by technology developers, regulators and researchers (Owen et al., 2012).

Making innovation processes more responsive implies that limited sociotechnical imaginaries and one-sided problem definitions need to be opened up for wider examination, by attending a plurality of future pathways (Pesch, 2018; Stirling, 2008). This demands that technology developers and regulators develop new approaches to organize innovation processes, which will allow for anticipating and reflecting on the consequences of innovation (cf. Guston, 2014). In turn, this requires the assessment of how credible and desirable the sociotechnical imaginaries that are implied by the promises about the new technology. Questions need to be posed about who is included and excluded in these imaginaries, about the problem definitions that support them, about how the forms of use that are intended. For network technologies, asymmetries of power and information need special attention, as the asymmetries tend to be reproduced by the self-reinforcing characteristics of these technologies.

A final point that needs to be raised here pertains to responsibilities of researchers in a domain that is characterized by performativity. As has been shown here, insights developed in research tend to spill over into the ‘real world’, as they become internalized in the mental models of actors involved in institutional and technological innovation. The notion of transaction costs is not only a descriptive label, but is also an explicit point of reference for technology development. An awareness about the interconnection between theory and practice necessitates the reconsideration of the epistemological and moral starting points of institutional economics, and should prompt theorists to develop new concepts and new frameworks to understand the challenges provided by technologies currently implemented and being implemented. The seamlessness, speed of development, and unavoidability of these new technologies make it necessary to reflect and act with much more caution than is practiced now.

## Notes

1. Though some authors see DL as a broader range of technologies than blockchain technologies, in this article we treat the terms as interchangeable.

2. Strictly speaking, blockchain technology enables key components for the implementation of decentralized markets – mediums of payment (cryptocurrencies) and arbitrage solutions (smart contracts). As we show, specific implementations can have different combinations of those elements. However, given that blockchain-based cryptocurrencies are the only mediums of decentralized exchange, it does not seem feasible to implement decentralized markets without blockchain elements at all.

3. At the moment of writing there are number of projects aiming to provide decentralized alternatives to established e-market platforms ranging from specialized to general purpose marketplace. See, e.g. Light (2019).

4. Currently, four cryptocurrency protocols are supported. See OpenBazaar.org (2019).

5. A Ricardian contract is essentially a cryptographically signed and verified digital contract which exists in two formats: one written in a plain text, and a copy written in a machine-readable format. This means that, unlike smart a contract, a Ricardian contract is not necessarily self-executable and not necessarily relies on a distributed computation. For an explanation on underlying technical differences and the project creators’ motivations on the choice of technology see Sanchez (2016).

6. This implementation is interesting in itself, since it essentially aims to provide decentralized alternative to the existing models of ‘programmatic advertising’ where ad content providers can acquire ad spaces via auction mechanisms. A crucial difference here is that unlike existing models this mechanism is not consumer identity centric but places contextual ads, avoiding thus the issue of consumers privacy.

7. OB1, the company behind OpenBazaar, has been involved in a somewhat controversial Bitcoin ‘civil war’ over the scaling proposal Segwit2x. One can argue that uncertainties associated with the struggle of institutional participants over Bitcoin protocol upgrades do introduce new costs for users of cryptocurrency and DMs, see Buntinx (2017).

8. ICOs are introduced as an analogy to the ‘initial public offering’ (IPO) of stocks. They are a first sale of cryptocurrency (or blockchain based tokens) to the public conducted for the purpose of fund raising.
